# From Nonobstructive Plaque to LAD Thrombosis in a 26-Year-Old Athlete

**DOI:** 10.1016/j.jaccas.2026.108124

**Published:** 2026-05-26

**Authors:** Mohammad Abu Zalam, Zeyad Kholeif, Usama Afzaal, Mustafa Dilawar Khan, Rahul Patri

**Affiliations:** Baptist Hospitals of Southeast Texas, Beaumont, Texas, USA

**Keywords:** anabolic steroid use, angiography, coronary thrombosis, endothelial dysfunction, plaque rupture, premature coronary artery disease

## Abstract

**Background:**

Stimulants and anabolic steroids have a role in plaque disruption resulting in acute coronary syndrome in young adults.

**Case Summary:**

A 26-year-old bodybuilder with heavy use of anabolic steroids and energy drinks presented with chest pain and ST-segment changes. Coronary angiography revealed minimal nonobstructive plaque in the proximal left anterior descending artery and left ventricular systolic dysfunction. He was started on guideline-directed medical therapy and counseled to discontinue toxic exposures. Weeks later, he presented with recurrent chest pain and was found to have an acute thrombotic occlusion of the mid–left anterior descending artery requiring urgent revascularization and a drug-eluting stent placement.

**Discussion:**

This case highlights how synergistic stimulant and anabolic exposure can create dynamic coronary pathology and contribute to endothelial injury, hypercoagulability, and accelerated atherosclerosis.

**Take-Home Messages:**

Nonobstructive coronary findings do not exclude imminent coronary thrombosis in high-risk young adults. Risk factor cessation and close follow-up are essential.

## Visual Summary

**Figure:**
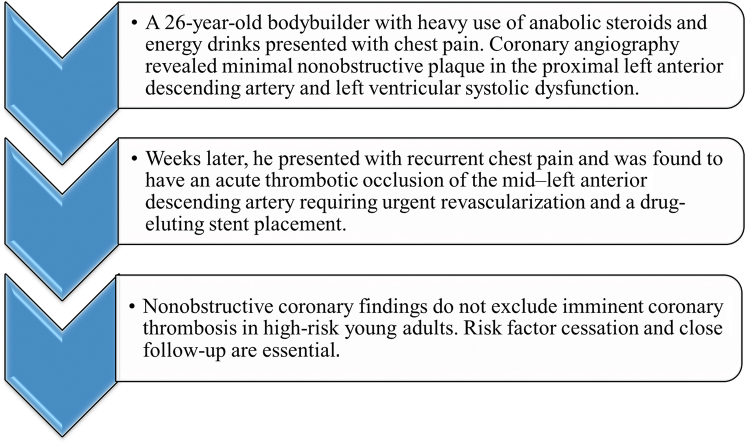

**Visual Summary:**
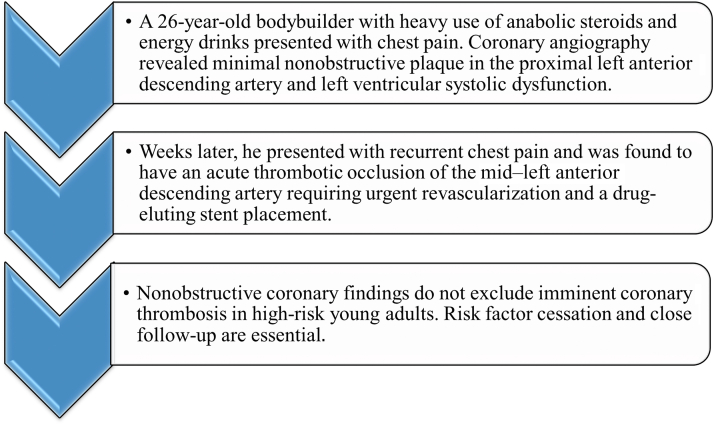
Progression From Nonobstructive Plaque to LAD Thrombosis

## History of Presentation

A 26-year-old man with no cardiovascular medical history or other significant comorbidities presented twice to our hospital within a span of 4 weeks with acute chest pain. On his first visit, he reported substernal pain radiating to the left shoulder with diaphoresis. The patient reported a history of long-term alcohol use, tobacco smoking (1 pack per day), excessive energy drink consumption, and anabolic steroid use (testosterone and trenbolone injections twice a week for the past 4 months). On physical examination, he appeared anxious and was in moderate distress. His cardiovascular examination revealed a regular rate and rhythm, with no murmurs or gallops. Cardiopulmonary examination was otherwise unremarkable.

**Take-Home Messages**

• Nonobstructive coronary findings can be misleading in high-risk young patients.
• Strict avoidance of cardiovascular stressors, along with close follow-up, is essential to mitigate recurrence risk.

## Investigations

His electrocardiogram (ECG) showed sinus tachycardia with a heart rate of 108 beats/min and deep T-wave inversions in the anterior leads (V_3_-V_6_) (Figure 1). Laboratory tests revealed elevated myocardial necrosis markers: creatine kinase 1514 U/L (reference value [RF] <170 U/L), troponin I 2.330 ng/mL (RF <0.033 ng/mL), creatine kinase–myocardial band 8.0 ng/mL (RF <2.4 ng/mL), and N-terminal pro–B-type natriuretic peptide 403 pg/mL (RF <100 pg/mL). Clotting factors were within normal limits. Given his ischemic symptoms, ECG abnormalities, and biomarker elevation, obstructive acute coronary syndrome (ACS) was initially suspected.

**Figure 1:**
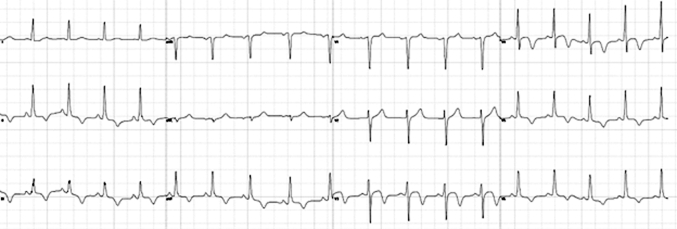
Electrocardiogram 1 The electrocardiogram displays sinus tachycardia with a heart rate of 108 beats/min and deep T-wave inversions in the anterior leads (V_3_-V_6_).

Urgent coronary angiography revealed right-dominant circulation with nonobstructive proximal left anterior descending artery (LAD) plaque and otherwise patent coronary arteries (Figure 2). The left ventricular end-diastolic pressure was 10 mm Hg, and the left ventriculogram showed an ejection fraction (EF) of 40% to 45% with apical and inferior wall hypokinesis. In the absence of flow-limiting stenosis, the presentation was approached within a myocardial infarction with nonobstructive coronary artery (MINOCA) framework. Coronary vasospasm was also considered in the setting of stimulant and tobacco exposure. Myocarditis and toxin-related cardiomyopathy were considered because of reduced EF and regional wall motion abnormalities; however, cardiac magnetic resonance imaging was not available at our facility and could have aided myocardial tissue characterization. In addition, intravascular imaging modalities such as intravascular ultrasound or optical coherence tomography, which may identify plaque rupture or erosion not apparent on angiography, were not available. No angiographic evidence of spontaneous coronary artery dissection was observed, and the ventricular dysfunction pattern was less typical for stress-induced cardiomyopathy.

**Figure 2:**
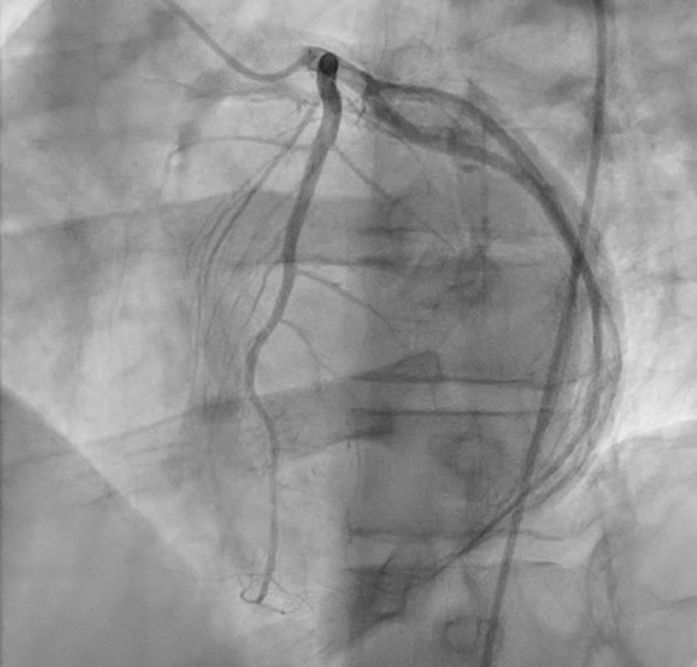
Coronary Angiogram 1 The initial coronary angiogram demonstrates a patent left anterior descending artery with mild nonobstructive proximal plaque and no flow-limiting stenosis.

## Management and Follow-Up

The patient was treated with dual antiplatelet therapy and guideline-directed medical therapy (GDMT) for left ventricular dysfunction and was counseled to discontinue anabolic steroids, stimulants, tobacco, and alcohol to avoid increased risk of plaque instability and rupture. He was discharged with prescriptions for metoprolol succinate (50 mg daily), losartan (25 mg daily), dapagliflozin (10 mg daily), spironolactone (25 mg daily), and atorvastatin (40 mg daily). At follow-up 1 week later, he reported cessation of anabolic steroid use, energy drink consumption, tobacco smoking, and alcohol intake, and adherence to prescribed medications.

Four weeks after discharge, the patient presented with crushing chest pain radiating to both arms, associated with nausea and shortness of breath. He reported that the pain started suddenly and was unrelenting, prompting him to seek urgent medical attention. A repeat ECG showed sinus rhythm with a heart rate of 96 beats/min and ST-segment elevations in the anterior leads (V_3_-V_6_), consistent with ST-segment elevation myocardial infarction (STEMI) (Figure 3). Given the recurrent chest pain and ECG findings, the patient was urgently taken to the cardiac catheterization laboratory for a second evaluation. Emergent angiography revealed complete thrombotic occlusion of the mid-LAD, with thrombus extending distally and an ulcerated proximal plaque (Figure 4). Aspiration thrombectomy followed by drug-eluting stent placement restored TIMI flow grade 3. Residual thrombus was noted in the apical cap, which was managed medically. Postprocedure ventriculography showed an EF of 30% to 35% with moderate diffuse hypokinesis. No other significant coronary artery disease was noted. The patient's ST-segment elevations completely resolved after the procedure.

**Figure 3:**
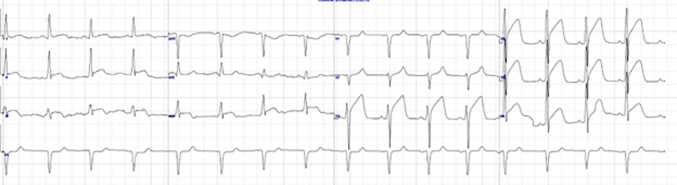
Electrocardiogram 2 The electrocardiogram displays sinus rhythm with a heart rate of 96 beats/min and ST-segment elevations in the anterior leads (V_3_-V_6_), consistent with ST-segment elevation myocardial infarction.

**Figure 4:**
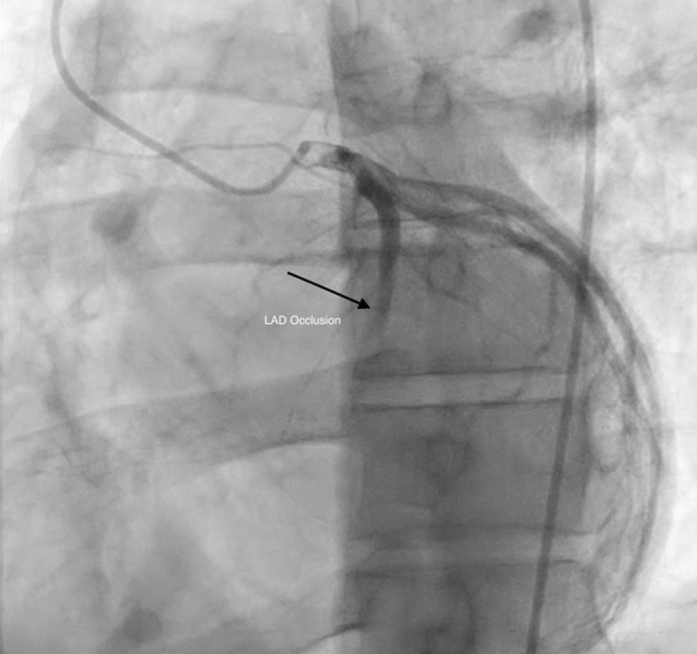
Coronary Angiogram 2 The coronary angiogram displays acute thrombotic occlusion of the mid–left anterior descending artery (LAD).

The patient was restarted on dual antiplatelet therapy with aspirin (81 mg daily) and clopidogrel (75 mg daily). He was continued on GDMT, including metoprolol succinate (50 mg daily), losartan (50 mg daily), spironolactone (25 mg daily), dapagliflozin (10 mg daily), and atorvastatin (40 mg daily). He was advised that cardiovascular risk may persist after cessation and that continued abstinence, medication adherence, and heart-healthy lifestyle modification are essential to reduce the risk of recurrent events.

## Discussion

This rare and interesting case illustrates how the combined use of anabolic-androgenic steroids (AAS), energy drinks, and unhealthy lifestyle factors contributed to myocardial injury and coronary thrombosis in a 26-year-old man. AAS misuse is a well-recognized cause of cardiomyopathy in otherwise healthy young athletes with long-term exposure and is associated with left ventricular hypertrophy, dilated cardiomyopathy (DCM), arrhythmias, and sudden cardiac death.^1^ Reports describe young bodybuilders with severe systolic dysfunction and normal or near-normal coronaries, often partially reversible after stopping AAS.^2^ This may explain our patient's initial reduced EF with regional hypokinesis despite nonobstructive coronary disease, suggesting AAS-related nonischemic DCM with superimposed myocardial injury.

AAS have proatherogenic and prothrombotic effects that predispose to premature coronary events. They cause direct myocyte toxicity, oxidative stress, interstitial fibrosis, and adverse remodeling. Chronic use is associated with an atherogenic lipid profile, endothelial dysfunction, increased platelet aggregation, erythrocytosis, and hypercoagulability.^1^ Case reports link AAS use in low-risk young adults to myocardial infarction from accelerated atherosclerosis and thrombosis, which may be missed on angiography when plaques are early or nonobstructive, as in our case.^3^^,^^4^ In addition, intravascular imaging and MINOCA data show many ACS events arise from nonobstructive lesions.^5^ Contemporary MINOCA frameworks emphasize systematic evaluation to distinguish plaque disruption, vasospasm, microvascular dysfunction, myocarditis, and other nonatherothrombotic causes of myocardial injury. The 2019 American Heart Association scientific statement further defines MINOCA as a working diagnosis requiring additional investigation to identify the underlying mechanism.^6^

Energy drinks add further cardiovascular risk, as their high caffeine and other stimulants can acutely raise blood pressure and heart rate, impair endothelial function, and enhance platelet activation.^7^ Case reports describe STEMI and coronary thrombosis in heavy energy drink users, sometimes without significant angiographic atherosclerosis. In our patient, chronic energy drink intake likely acted synergistically with AAS, cigarette smoking, and alcohol to trigger plaque disruption or endothelial injury and in situ thrombosis.^7^^,^^8^

In our patient, management was extrapolated from standard DCM and STEMI guidelines, as no specific recommendations exist for anabolic steroid– or energy drink–related cardiomyopathy. His left ventricular systolic dysfunction was treated with GDMT (angiotensin-converting enzyme inhibitor/angiotensin receptor blocker or sacubitril-valsartan, β-blocker, mineralocorticoid receptor antagonist, and sodium-glucose cotransporter 2 inhibitor), with plans to continue these agents despite potential EF recovery, consistent with GDMT for heart failure with reduced EF.^9^ After he developed acute LAD thrombosis, care followed STEMI protocols with urgent revascularization, dual antiplatelet therapy, and high-intensity statin, alongside ongoing left ventricular dysfunction therapy.^9^ Equally important were aggressive risk factor modification, and that included cessation of anabolic steroids, energy drinks, tobacco, and alcohol and close clinical and echocardiographic follow-up to monitor ventricular recovery and recurrent ischemic risk.^9^

In our case, the overall course is best explained by a multihit model: chronic anabolic steroid exposure and alcohol use likely contributed to myocardial injury and left ventricular dysfunction on a background of subclinical or nonobstructive coronary disease. Despite reported cessation of toxic exposures, endothelial dysfunction and plaque vulnerability may have persisted, allowing a previously unapparent LAD lesion to undergo plaque erosion or rupture with superimposed thrombus, presenting as anterior STEMI with angiographic occlusion, for which he received standard ACS and heart failure management.

### Limitations

This report has several limitations. Cardiac magnetic resonance imaging and intravascular imaging (intravascular ultrasound or optical coherence tomography) were not available at our institution, limiting myocardial tissue characterization and definitive assessment of plaque rupture or erosion. A comprehensive hypercoagulable evaluation was not performed. Abstinence from toxic exposures was self-reported and could not be independently verified. As a single-patient observation, these findings may not be generalizable.

## Funding Support and Author Disclosures

The authors have reported that they have no relationships relevant to the contents of this paper to disclose.
